# Hepatitis e prevalence, knowledge, and practice of preventive measures among secondary school adolescents in rural Nigeria: a cross-sectional study

**DOI:** 10.1186/s12889-021-11702-y

**Published:** 2021-09-10

**Authors:** Chioma Ngozichukwu Pauline Mbachu, Joy Chinelo Ebenebe, Henry Chima Okpara, John Onuora Chukwuka, Ikechukwu Innocent Mbachu, Jacinta Chinyere Elo-Ilo, Chizalu Ifeyinwa Ndukwu, Ifeoma Egbuonu

**Affiliations:** 1 Department of Paediatrics, Nnamdi Azikiwe University, Awka, Anambra State Nigeria; 2 Department of Chemical Pathology, Nnamdi Azikiwe University, Awka, Anambra State Nigeria; 3 Department of Obstetrics and Gynaecology|, Nnamdi Azikiwe University, Awka, Anambra State Nigeria; 4 Department of Paediatrics, Chukwuemeka Odumegwu Ojukwu University, Awka, Anambra State Nigeria

**Keywords:** Hepatitis E, Adolescents, Prevalence, Knowledge, Practice, Nigeria

## Abstract

**Background:**

Currently, there is a paucity of data on the knowledge and practice of preventive measures for Hepatitis E infection in Nigerian populations. This study provided data on the prevalence, knowledge and practices of prevention in an adolescent population from Nigeria.

**Methods:**

This cross-sectional study was conducted over 3 months among rural Nigerian secondary school adolescents. An interviewer-based questionnaire was used to collect data on sociodemographic profile, knowledge, and practice of preventive measures for Hepatitis E infection. Blood samples collected from participants were analysed for Hepatitis E IgG using Elisa Kits (Sigma Diagnostics, USA). Data were analysed using SPSS software version 20.0. Tests of association were done with a level of significance set at 5%.

**Results:**

A total of 9 out of the 414 participants tested positive for Hepatitis E IgG antibodies giving a prevalence of 2.2%. Significant factors for Hepatitis E infection were male gender {*P* = 0.004} and school {*P* < 0.001, however logistic regression gave infinite value. Most participants (98.6%) had poor knowledge of Hepatitis E infection, 239(57.7%) had good preventive practices, while 175(42.3%) had average preventive practices.

**Conclusion:**

A low prevalence of HEV infection was recorded among study participants. There was poor knowledge of Hepatitis E, and association could not be established between HEV infection and individual preventive practices.

**Supplementary Information:**

The online version contains supplementary material available at 10.1186/s12889-021-11702-y.

## Background

Globally, 20.1 million people are estimated to have Hepatitis E infection every year. Of these, 3.4 million become symptomatic, with mortality being as high as 70,000, and 3000 stillbirths annually [1]. About a third of the world’s population (2.2 billion) comprises children and adolescents [2]. Hepatitis E infection has been documented as the most common cause of viral Hepatitis with higher prevalence in adolescents and young adults [3–5]. A high prevalence rate of 75.6 and 75.5% was observed among adolescents aged 10–14 years and 15–19 years respectively in a study done among 5112 healthy children in Egypt by Fix et al. [6]

The concern about the potential long term complications has made its screening to be part of the screening guidelines for blood product transfusion in some developed countries [7]. This has also led to a growing call for the Hepatitis E vaccine based on the disease burden. Several works have been done worldwide on Hepatitis E infection but in Nigeria, there is a paucity of data [8–13].

Hepatitis E infection is an evolving disease with several risk factors, but the distribution varies depending on the population (rural or urban), sociodemographic factors, provision of clean water, food /hygiene practices, sewage disposal, and health status [8]. Direct person-to-person transmission via the faecal–oral route has been suggested as an additional factor contributing to both epidemic and sporadic cases of HEV [14].

In developing countries, HEV infection is a public health problem causing both sporadic and epidemic cases that affects several thousands of human lives [15, 16]. Since there is no cure for Hepatitis E infection and vaccines are not readily available, the main strategy for reducing the disease burden is the prevention of the infection. Most studies done focused mainly on the prevalence, while some documented the risk factors [8]. There is a paucity of data on the knowledge and practice of preventive measures for Hepatitis E infection. Determination of knowledge and practice of preventive measures are necessary for informed health education to reinforce positive habits and discourage negative habits that predispose people to Hepatitis E infection and other communicable diseases. This study determined the prevalence of HEV in a population of adolescents in South-East Nigeria. This study also assessed knowledge of HEV in the study population as well as identified preventative measures to reduce infection risk.

## Methods

The study was a cross-sectional descriptive study conducted from April to July 2017, among secondary school students in Anambra State, South-East Nigeria. Only secondary school students whose ages are from 10 to 18 years who had resided in Anambra state for at least 2 months (incubation period of HEV) were recruited for the study. The school authorities and class teachers were informed about the study before its commencement. Each parent/guardian of participants less than 18 years old signed the informed consent document for his/her child/ward. Assent was given by participants less than 18 years old. The informed consent form was delivered to the parents/guardians through prospective participants.

The sample size of 371 was calculated using the formula [17] *n* = $\frac{{\mathrm{Z}}^2\mathrm{P}\left(1-\mathrm{P}\right)}{{\mathrm{d}}^2}$, where n is the minimum sample size, Z is the normal standard deviate corresponding to a significance level. For a significance level of 3%, Z = 2.17, using the area under normal distribution curve.

P is the proportion of outcome of interest recorded by Ekanem et al [9] in Nigeria which is 0.077; d is the maximum likely error = 3% = 0.03. Assuming a non-response rate of 20%, sample size of 465 was calculated.

### Sampling technique

A Multi staged sampling technique was used to recruit participants for the study. Firstly, schools were selected using a stratified sampling technique to ensure appropriate representation of participants (public and private; same gender and co-educational schools; class, males and females). Secondly, stratified sampling was used to select participating classes. Each school is already stratified into 6 classes namely JSS1, JSS2, JSS3, SS1, SS2 and SS3 from where study participants were selected. For classes that have arms like JSS1A, JSS1B, JSS1C, simple random sampling technique was used to select the representative class in each set.

Thirdly, simple random sampling was employed in selecting participants from each class using the class register as a sampling frame. A total of 6 schools were used for the study. The total population of students in the 21 private schools was 1212, while that of students in the 8 public schools was 1117 giving a ratio of approximately 1:1(1.1:1).

School A is a private, boarding, boys’ only school while School B is a public boarding/day boys’ only school. School C is a public, day co-educational school and School D is a private day, co-educational school. School E is girls’ only private boarding school while School F is a girls-only public boarding school.

From school A, 76 students were studied, while 70 students were studied in school B, 85 students in school C, 66 students in school D, 76 students in school E, and 41 students in school F. This selection was based on the population of the schools. Participants that opted out of the study from the six schools were replaced by simple random sampling from the classes in the schools.

### Study instruments and materials

A semi-structured interviewer-administered questionnaire that was pretested in a school that was not part of the study was used for data collection (supplementary file). The information included socio-demographic profile, awareness of Hepatitis including routes of transmission, symptoms, personal hygiene factors, social practices, and preventive practices against Hepatitis E. A study identification number was assigned to each subject and this identification number was used to label the specimen bottles. At the end of the study, there was health education on symptoms, signs, modes of transmission, treatment options, and preventive measures for viral hepatitis with emphasis on Hepatitis E infection.

#### Laboratory procedure

The study was done using ELISA test kits by Sigma diagnostics, USA (Lot Number: JG7190–12) for the detection of IgG to Hepatitis E virus in human serum. The sensitivity and specificity are both > 98%. Sera from all participants were tested for seroreactivity to anti-HEV IgG antibodies (ELISA kits by Sigma Diagnostics, USA). Positive seroreactivity anti-HEV IgG antibodies indicated a previous/past HEV infection. Positive and negative control specimens that were obtained from the SIGMA ELISA kits were run concurrently with the sera during respective batch analysis for anti-HEV IgG antibodies. They were used to assess the validity of the immunoassay procedure and ensure the analytical quality of the ELISA procedure. Test results were interpreted as a ratio of the absorbance of the sample (As) and the cut-off absorbance (Ac). AS/AC < 0.200 implied a negative result, AS/AC between 0.200 and 0.500 was indeterminate, while AS/AC > 0.500 was positive. A negative result indicated that the participant had not been infected by HEV. A positive result was indicative of previous HEV infection. Test results were disclosed to each participant and their parents/guardians.

### Knowledge assessment

Knowledge was assessed by summing responses to questions on awareness (maximum of 1 point), routes/modes of transmission (maximum of 4 points), and symptoms/signs (maximum of 5 points), giving a maximum score of 10 points. Scores of 0–4 (< 40%) were adjudged as poor knowledge, 5–6 (50–60%) was average knowledge, while 7–10 (> = 70%) was good knowledge. The practice of preventive measures for Hepatitis E (7 items) was scored by the summation of responses to questions on preventive measures, giving a maximum of 12 points. Questions on Hand wash before meals, Hand wash after toilet use, and Fruit and vegetable wash before consumption, were scored using a 2-point Likert scale (1 = sometimes, 2 = always), while the question on eating from a common plate was scored using a reverse Likert 3-point scale (1 = always, 2 = sometimes, 3 = never). The other responses under the Practice of preventive measures (Consumption of wild game meat, alcohol consumption, and intravenous drug use) scored a maximum of 1 point each. Scores of 0–5 (< 41%) were categorized as poor practice, 6–8 (50–67%) were scored as average practice, while 9–12(> = 75%) were adjudged as good practice.

#### Data entry and analysis

All completed questionnaires and proforma were coded before entry into IBM SPSS Statistics software version 20.0 for Windows which was used for analysis. Quantitative variables like age, knowledge and practice of preventive measures were categorized before analysis. Age was categorized into 10–13 years (early adolescence), 14–16 years (middle adolescence) and > 16 years (late adolescence) [18]. Knowledge and practice scores were categorized into poor, average and good respectively based on the summation of the scores. Categorical variables were summarized using frequencies and percentages. Cross-tabulated categorical variables {Age group, sex, class, social class (Oyedeji’s classification) [19], seroprevalence, student status, personal hygiene factors, knowledge scores, practice scores} were tested for association using Pearson’s Chi-square (and Fisher’s exact test where appropriate) at a statistically significant *P*-value of < 0.05, providing a confidence interval of 95%. The dependent variable for the analysis was the presence or absence of Hepatitis E infection. The independent variables were the socio-demographic factors, knowledge, and practice of Hepatitis E infection preventive measures.

Ethical approval for the study was obtained from the Ethics and Research committee of Nnamdi Azikiwe University Teaching Hospital, Nnewi (NAUTH/CS/66/VOL.9/154/2016/127). Permission was obtained from the Post Primary Schools Board, Nnewi zone, the State Ministry of Education (MOE/SCHD/1570/VOL.I/64), and the principals of the schools. The guidelines on research involving the use of human subjects were strictly adhered to according to the Helsinki Declaration. Participants did not incur any cost by participating in this study and there was no financial inducement.

## Results

**Fig. 1:**
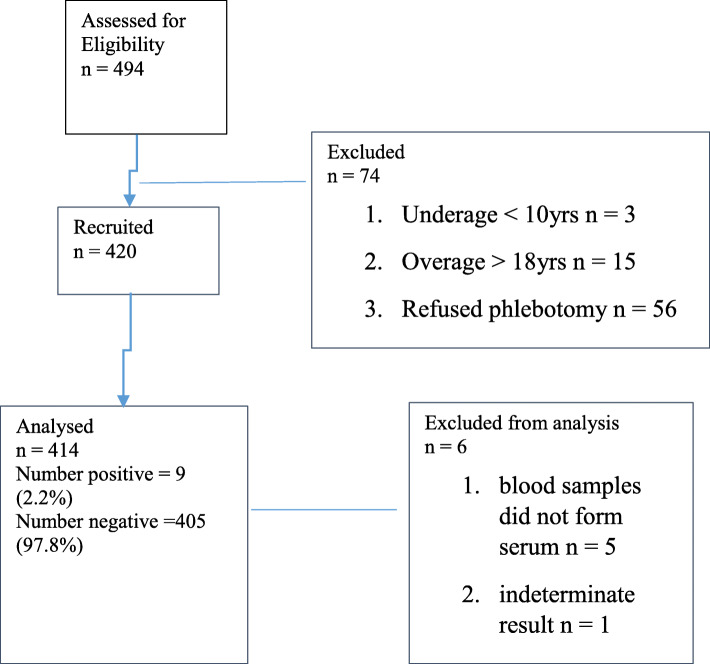
Flowchart for participants

Four hundred and twenty adolescents participated in the study. However, only 414 participants who had determinate results were included in the final analysis. Five samples whose sera could not be separated and one indeterminate result was excluded (Fig. 1 shows the flow chart of participants).

About half of the participants 220(53.1%) were males, and almost half 218(52.7%) were junior secondary school students. Mean age of participants was 14.5 ± 1.8 years. Two hundred and twenty-three students (53.9%) comprised the 14–16-year age group. Day students comprised 213(51.5%) of the study population, while there were 201(48.5%) boarding students. The lower socioeconomic class accounted for 215(51.9%) of the participants.

Of the 414 participants’ sera analyzed, 9 were positive for HEV IgG giving a seroprevalence rate of 2.2%. All participants who had positive seroreactivity were males. All anti-HEV seropositive participants attended school B. There was a significant association between gender and HEV infection {*P* = 0.004}. However, bivariate analysis showed infinite results. There was no significant association between age group or socioeconomic class and Hepatitis E infection {*P* > 0.05}. Table 1 shows the relationship between sociodemographic parameters and anti-HEV IgG.

**Table 1:** Relationship between Sociodemographic parameters and anti-HEV IgG

VARIABLE	FREQUENCY (***N*** = 414)	HEPATITIS E		***P*** - VALUE
		POSITIVE (%)	NEGATIVE (%)	
**AGE GROUP (years)**				
10–13	141	4 (2.8)	137 (97.2)	0.633
14–16	223	5 (2.2)	218 (97.8)	
> 16	50	0 (0.0)	50 (100.0)	
**CLASS**				
Junior secondary	218	6 (2.8)	212 (97.2)	0.509
Senior secondary	196	3 (1.5)	193 (98.5)	
**SEX**				
Male	220	9 (100.0)	211 (95.9)	0.004*
Female	194	0 (0.0)	194 (100.0)	
**SCHOOLS USED**				
A	76	0 (0.0)	76 (100.0)	< 0.001*
B	70	9 (12.9)	61 (87.1)	
C	85	0 (0.0)	85 (100.0)	
D	66	0 (0.0)	66 (100.0)	
E	76	0 (0.0)	76 (100.0)	
F	41	0 (0.0)	41 (100.0)	
**SOCIOECONOMIC**				
**CLASS**				
Upper	74	0 (0.0)	100 (100.0)	0.407
Middle	125	3 (2.4)	122 (97.6)	
**STUDENT STATUS**				
Day	213	7 (3.3)	206 (96.7)	0.177
Boarding	201	2 (1.0)	199 (99.0)	
Total	414	9 (2.2)	405 (97.8)	

A- Private, boarding, boys’ only school, pit latrine use, borehole * = significant *P*-value

B- Public, boarding/day, boys’ only school, pit latrine use, borehole

C- Public, day, co-educational, open defecation, sachet/ water from home

D- Private, day, co-educational, pit latrine use, sachet/water vendor/ home

E- Private, boarding, girls’ only, pit latrine use, sachet/borehole

F- Public, boarding, girls’ only, water cistern, borehole

Most participants (98.6%) had poor knowledge of HEV infection compared to 6/414(1.4%) who had average knowledge. No participant had good knowledge. There was no significant association between knowledge summative score and HEV infection (*p* = 1.000). Of the 239/414(57.7%) students who had good practices of HEV preventive measures, 2/239 (0.8%) had anti-HEV IgG seropositivity compared to 7/175 (4.0%) seropositivity among participants who had average practice. Table 2 shows participants’ knowledge and preventative practices summative score for HEV infection.

**Table 2:** Participants’ Knowledge and Preventative Practices summative score for Hepatitis E infection

PARAMETER	FREQUENCY (%)	HEPATITIS E		*P*- VALUE
		Positive	Negative	
KNOWLEDGE				
Average	6 (1.4)	0.0 (0)	6 (100.0)	1.000
Poor	408 (98.6)	9 (2.2)	399 (97.8)	
PRACTICE				
Good	239 (57.7)	2 (0.8)	237 (99.2)	0.040*^#^
Average	175 (42.3)	7 (4.0)	168 (96.0)	
TOTAL	414	9 (2.2)	405 (97.8)	

* = significant *P*-value

# = Fisher’s exact value

Only 37/414 (8.9%) participants were aware of Hepatitis E infection. Four participants (1.0%) were aware of its transmission through the waterborne route, while 25/414 (6.0%) knew it could be transmitted through blood. None was aware of its spread through either food or direct contact. Twenty-two participants (5.3%) identified yellowness of the eyes as a symptom of HEV infection, while only 10/414 (2.4%) mentioned fever as a symptom. Table 3 shows the Relationship between Hepatitis E Knowledge and HEV infection among participants.

**Table 3:** Relationship between Hepatitis E knowledge and HEV infection among participants

PARAMETER	FREQUENCY (%)	HEPATITIS E		*P*-value
		Yes (%)	No (%)	
AWARENESS				0.615^b^
Yes	37 (8.9)	0 (0.0)	37 (100.0)	
No	377 (91.1)	9 (2.4)	368 (97.6)	
ROUTES/MODES OF TRANSMISSION				
Waterborne				1.000^b^
Yes	4 (1.0)	0 (0.0)	4 (100.0)	
No	410 (99.0)	9 (2.2)	401 (97.8)	
Food Borne				0.626 ^b^
Yes	0 (0.0)	0 (0.0)	0 (0.0)	
No	414 (100.0)	9 (2.2)	405 (97.8)	
Blood Borne				1.000 ^b^
Yes	25 (6.0)	0 (0.0)	25 (100.0)	
No	389 (94.0)	9 (2.3)	380 (97.7)	
Direct contact				1.000 ^b^
Yes	0.0 (0.0)	0 (0.0)	0 (0.0)	
No	414 (100.0)	9 (2.2)	405 (97.8)	
SYMPTOMS				
Yellowness of eyes				1.000 ^b^
Yes	22 (5.3)	0 (0.0)	22 (100.0)	
No	392 (94.7)	9 (2.3)	383 (97.7)	
Fever				1.000 ^b^
Yes	10 (2.4)	0 (0.0)	10 (100.0)	
No	404 (97.6)	9 (2.2)	395 (97.8)	
Weakness				1.000 ^b^
Yes	8 (1.9)	0 (0.0)	8 (100.0)	
No	406 (98.1)	9 (2.2)	397 (97.8)	
Headache				1.000 ^b^
Yes	7 (1.7)	0 (0.0)	7 (100.0)	
No	407 (98.3)	9 (2.2)	398 (97.8)	
Total	414 (100.0)	9 (2.2)	405 (97.8)	

b = Fisher’s Exact value

On preventive practices towards HEV infection, 160/414(38.6%) participants “always” washed their hands before meals compared to 254/414 (61.4%) participants who washed “sometimes” before meals. Two hundred and twenty-four students (54.1%) always washed hands after toilet use compared to 190/414 (45.9%) who washed hands “sometimes”. Only 141/414(34.1%) participants ate wild game meat compared to 273/414(65.9%) who did not. Four participants (1.0%) used illicit intravenous drugs. Individually, none of the risk factors was significantly associated with HEV infection (Table 4).

**Table 4:** Relationship between Hepatitis E preventive practices and HEV infection among participants

PARAMETER	FREQUENCY (%)	HEPATITIS E		*P*-VALUE
		Yes (%)	No (%)	
**Hand wash before meals**				1.000 ^b^
Sometimes	254 (61.4)	3 (1.2)	251 (98.8)	
Always	160 (38.6)	6 (3.8)	154 (96.2)	
**Hand wash after toilet use**				0.087
Sometimes	190 (45.9)	7 (3.7)	183 (96.3)	
Always	224 (54.1)	2 (0.9)	222 (99.1)	
**Fruit/vegetable wash before consumption**				0.316 ^b^
Sometimes	242 (58.5)	7 (2.9)	235 (97.1)	
Always	172 (41.5)	2 (1.2)	170 (98.8)	
**Use of common plate**				0.095 ^b^
Always	60 (14.5)	3 (5.0)	57 (95.0)	
Never	95 (22.9)	6 (2.3)	253 (97.1)	
Sometimes	259 (62.6)	0 (0.0)	95 (100.0)	
**Wild game meat consumption**				0.284 ^b^
Yes	141 (34.1)	5 (3.6)	136 (96.4)	
No	273 (65.9)	4 (1.5)	269 (98.5)	
**Alcohol consumption**				0.423 ^b^
Yes	92 (22.2)	3 (3.3)	89 (96.7)	
No	322 (77.8)	6 (1.9)	316 (98.1)	
**Intravenous drug use**				0.084 ^b^
Yes	4 (1.0)	1 (25.0)	3 (75.0)	
No	410 (99.0)	8 (1.9)	402 (98.1)	
Total	414 (100.0)	9 (2.2)	405 (97.8)	

b = Fisher’s Exact value

## Discussion

The seroprevalence of Hepatitis E infection was 2.2% in this study. This seroprevalence rate is lower than 6.5% recorded by Bugaje *et al* [10] among secondary school students in Kaduna state, North-west Nigeria, 7.7% by Ekanem *et al* [9] among children in Cross River State, South-south Nigeria; 13.4% by Adesina *et al* [20] in Ekiti state, southwestern Nigeria. The low prevalence noted in this study could also be because of the homogenous group of in-school adolescents studied. The rate among out-of-school adolescents may be different and is worth exploring.

The variations observed in the different studies in Nigeria may be related to the characteristics of the study population which include age, urban versus rural area, gender, sick or healthy population, the study design, presence, and absence of a clinical and subclinical outbreak, and other risk factors.

The age of participants in this study did not influence seroprevalence. This was contrary to reports by Ekanem *et al* [9], Bugaje *et al* [10] and Junaid *et* al [21] that had observed a significant association between the age of study participants and HEV infection. Different studies that had reported a significant association between age and HEV infection had a wide age range of participants (from 0 to 20 years and beyond) [5, 9, 10, 21]. Participants in the study by Ekanem *et al* [9] were between 1 and 18 years old while the age of study participants in the study by Bugaje *et al* [10] ranged from 11 to 20 years.

Only males had anti-HEV IgG seropositivity but logistic regression showed infinite value thus the association of HEV and gender could not be ascertained. However, previous studies by Bugaje *et al* [10] and some scholars in the systematic review by WHO [8] demonstrated that male gender is more commonly associated with HEV infection. The reasons for this higher prevalence among males are unclear as reported by several authors but it has been postulated that males tend to indulge in risky behaviour such as alcohol ingestion, eating out in food vendors’ or retailers’ stalls, and sexual debut [22]. However, Martinson *et al* [12] in Ghana observed a higher prevalence among females. The reason stated was that in rural Ghana, women are more involved in fetching water from streams, taking care of young children, and thus more likely to be exposed to faeco – oral contamination.

All participants who were HEV positive attended the same school which was a public, exclusive male school, had both boarding and day students, students shared the same toilet facility type at school (pit latrine) and possibly drank from the same water source (borehole water supply). Although there was no significant relationship between HEV and sources of drinking water, the sources of water and purity of these sources may differ among different localities. Thus, it may be necessary to explore the sources of drinking water in schools and communities for possible contamination, in view of the possible cluster of HEV among these students. No significant association was found between socioeconomic class and HEV infection, similar to findings by Ekanem *et al* [9] in Cross-river state, Nigeria, and Buti *et al* [23] in Spain that observed no as.

sociation between social class and HEV infection. This could be because most students in this study had similar sources of drinking water and sewage disposal methods irrespective of their social class.

The study showed poor awareness, knowledge of symptoms, and modes of transmission of HEV infection. This may be because Hepatitis E infection is an emerging disease that is not well known in the rural areas. Thus, the need for periodic health education in schools to improve the awareness of HEV infection and other communicable diseases. There was no significant association between knowledge and prevalence of Hepatitis E. There was a good practice of preventive measures among participants, however, the individual risk factors were not independently associated with the prevalence of Hepatitis E infection.

Our study assessed the association between HEV infection, practice of preventive measures and knowledge of HEV infection. This is one of the few studies that tried to find an association between knowledge, practice of preventive measures and HEV infection. Previous studies focused mainly on the prevalence while few others studied the risk factors. The result of this study showed that the participants had poor knowledge. Thus, there is need to raise awareness of this emerging disease among the adolescents especially because of the potential for sporadic outbreaks. The study did not find strong association between the rate of infection and preventive practices. This may be due to the low prevalence of the HEV infection recorded in this study. In addition, participants shared similar sociodemographic characteristics, which may have contributed to the observed result. Further studies including people of different sociodemographic characteristics will be needed to ascertain or refute this observation.

Conclusion/Recommendation: A low prevalence of HEV infection was recorded among study participants, who also demonstrated poor knowledge of HEV infection, but good or average preventive practices. We infer that HEV infection rate is low among healthy adolescents. The inability of the study to demonstrate a significant association between HEV infection, gender and school due to infinite nature of the data calls for further studies. Future studies may incorporate adolescents with compromised immune conditions and out of school adolescents.

The observations of this study should be interpreted with caution. This is because most of the participants are healthy in-school adolescents. Thus, it should not be generalized to immune-compromised and out-of-school adolescents.

## Supplementary Information

**Additional file 1.**

## Data Availability

The datasets used and/or analysed during the current study are available from the corresponding author on reasonable request.

## Abbreviations

Anti-HEV: Antibody to Hepatitis E virus
HEV: Hepatitis E virus
IgG: Immunoglobulin G

## References

[1] WHO. Hepatitis E vaccine: WHO position paper, May 2015 – Recommendations. Vaccine. 2015. 10.1016/j.vaccine.2015.07.056. 10.1016/j.vaccine.2015.07.056 26232546

[2] UNICEF (2017). The state of the world’s children: Statistical tables.

[3] Purcell RH, Emerson SU (2008). Hepatitis E: An emerging awareness of an old disease. J Hepatol.

[4] Khuroo MS, Khuroo MS (2016). Hepatitis E: An emerging global disease - from discovery towards control and cure. J Viral Hepat.

[5] Verghese VP, Robinson JL (2014). A systematic review of hepatitis E virus infection in children. Clin Infect Dis.

[6] Fix AD, Abdel-hamid M, Purcell RH, Shehata MH, Abdel-aziz F, Mikhail N (2000). Prevalence of antibodies to hepatitis E in two rural Egyptian communities. Am J Trop Med Hyg.

[7] Joint UKBTS Professional Advisory Committee (2015). Change Notification UK National Blood Services No. 29–2015. NHS Blood Transpl.

[8] WHO. The Global Prevalence of Hepatitis E Virus Infection and Susceptibility: A Systematic Review: World Health; 2010. Available from: http://www.who.int/iris/handle/10665/70513.

[9] Ekanem E, Ikobah J, Okpara H, Udo J (2015). Seroprevalence and predictors of hepatitis e infection in Nigerian children. J Infect Dev Ctries.

[10] Bugaje M, Balogun S, Abdulkadir I, Ahmed A (2016). Seroprevalence of Hepatitis E infection among secondary school students in Kaduna State, Northern Nigeria. Sahel Med J.

[11] Asaei S, Ziyaeyan M, Moeini M, Jamalidoust M, Behzadi MA (2015). Seroprevalence of Hepatitis A and E virus infections among healthy population in Shiraz, Southern Iran. Jundishapur J Microbiol.

[12] Martinson FE, Marfo VY, Degraaf J (1999). Hepatitis E virus seroprevalence in children living in rural Ghana. West Afr J Med.

[13] Dong C, Dai X, Liang J, Dong M, Meng J (2012). Seroprevalence of hepatitis e virus varies considerably among Chinese provinces. Hepat Mon.

[14] Teshale EH, Grytdal SP, Howard C, Barry V, Kamili S, Drobeniuc J, Hill VR, Okware S, Hu DJ, Holmberg SD Evidence of person-to-person transmission of hepatitis E virus during a large outbreak in northern Uganda. Clin Infect Dis 2010;50(7):1006–1010. Available from: 10.1086/651077 10.1086/651077 20178415

[15] Kamar N, Bendall R, Legrand-Abravanel F, Xia NS, Ijaz S, Izopet J, Dalton HR (2012). Hepatitis e. Lancet.

[16] WHO (2017). Acute hepatitis E.

[17] Lemeshow S, Hosmer DW, Klar J, Lwanga SK, Lemeshow S, World Health Organization (1990). Adequacy of sample size in health studies. Part 1: Statistical methods for sample size determination.

[18] Fatiregun AA, Kumapayi TE. Prevalence and correlates of depressive symptoms among in-school adolescents in a rural district in Southwest Nigeria. J Adolesc 2014;37(2):197–203. Available from: 10.1016/j.adolescence.2013.12.003 10.1016/j.adolescence.2013.12.003 24439625

[19] Oyedeji GA (1985). Socio-economic and cultural background of hospitalized children in Ilesha. Niger J Paediatr.

[20] Adesina OA, Japhet MO, Donbraye E, Kumapayi TE, Kudoro A (2009). Anti hepatitis E virus antibodies in sick and healthy children in Ekiti state. Afr J Microbiol Res.

[21] Junaid SA, Agina SE, Abubakar KA (2014). Epidemiology and associated risk factors of hepatitis E virus infection in plateau state, Nigeria. Virol Res Treat.

[22] Marano G, Vaglio S, Pupella S, Facco G, Bianchi M, Calizzani G, Candura F, Catalano L, Farina B, Lanzoni M, Piccinini V, Liumbruno GM, Grazzini G (2015). Hepatitis E: an old infection with new implications. Blood Transfus.

[23] Buti M, Plans P, Domínguez A, Jardi R, Rodriguez Frias F, Esteban R (2008). Prevalence of hepatitis E virus infection in children in the northeast of Spain. Clin Vaccine Immunol.

