# Supplementary material for: Hepatitis e prevalence, knowledge, and practice of preventive measures among secondary school adolescents in rural Nigeria: a cross-sectional study
Source: BMC Public Health. 2021 Sep 10;21:1655. doi: 10.1186/s12889-021-11702-y (PMC8434741; doi:10.1186/s12889-021-11702-y)
Supplement: Supplementary file 1 — Additional file 1. [file 12889_2021_11702_MOESM1_ESM.docx]

**QUESTIONAIRE**

This questionnaire is to help determine PREVALENCE, RISK FACTORS, KNOWLEDGE OF HEPATITIS E VIRUS INFECTION AND PREVENTIVE PRACTICES AMONG SECONDARY SCHOOL STUDENTS IN ANAMBRA STATE, NIGERIA

All information in this questionnaire will be treated confidentially.

**INSTRUCTIONS**

Please read the questions carefully and then fill in the responses as accurately as possible. If you are unsure, call my attention for an explanation.

**SECTION A: SOCIO DEMOGRAPHIC DATA**

1. Subjects’ code number----------------------
2. Date:-------------------------
3. Age at last birthday--------------------
4. Ethnicity (a) Igbo (b) Yoruba (c) Hausa (d) others (specify)---------------------------
5. Class----------------------
6. Mode: (a) Day student (b.) Boarding

**SECTION B: PERSONAL HYGIENE**

1. How often do you wash your hands after going to the toilet? a) always b) Sometimes c) Never
2. How often do you wash your hands before eating? a) always b) Sometimes c) Never
3. How often do you wash fruits and vegetables before eating? (a) Always (b) Sometimes (c) never

**SECTION C: MEDICAL HISTORY**

1. Have you been screened for Hepatitis before? (a) Yes (b) No
2. If yes, the result was? (a) Negative (b) Positive (c) Not known
3. Any history of jaundice a) Yes b) No
4. If yes when?-----------------
5. Have you had a blood transfusion? (a) Yes (b) No
6. If yes, when?-----------
7. If yes, for what?--------------
8. Are there any drugs you take every day or regularly for any condition?
9. What are those drugs?
10. Have you ever been tested for HIV? (a) Yes (b) No
11. If yes, what was the result? (a) positive (b) negative (c) Do not Know
12. Do you have Diabetes/sugar disease? (a) Yes (b) No (c) Do not Know.
13. Have you ever had surgery a) Yes b) No
14. If yes, when?------ For what?------
15. Have you ever had dental extractions a) Yes b) No
16. Have you ever had accidents causing bleeding wounds? a) Yes b) No
17. Have you also helped people or intervened during accidents? a) Yes b) No
18. What is your genotype? a) AA b) AS c) SS d) don’t know

**SECTION D: FAMILY AND SOCIAL HISTORY**

1. Educational qualification of the Mother: (a) Tertiary (b) Secondary (c) Primary (d) No formal Education.
2. Educational qualification of the Father: (a) Tertiary (b) Secondary (c) Primary (d) No formal Education.
3. Occupation of Father----------------------------------
4. Mother’s Occupation………………………….
5. Social class (using Oyedeji’s classification)…………………………………..
6. Source of Drinking Water at home (a) stream (b) borehole (c) rain water (d) water vendor (e) sachet water (f) bottled water
7. Source of drinking water at School (a) stream (b) borehole (c) rain water (d) water vendor (e) sachet water (f) bottled water
8. How many students are in your class?-------------------
9. Type of accommodation at home (a) single bedroom apartment (b) 2 bedroom flat (c) 3 or more bedrooms
10. How many of you sleep together in a room? ---------
11. How many of you sleep on the same bed/mat?----------------
12. Do you eat from a common plate? (a) Always (b) Sometimes (c) never
13. Type of toilets in school (a) pit latrine (b) water closet (c) bucket (d) none.
14. Type of toilets at home (a) pit latrine (b) water closet (c) bucket (d) none
15. Do you have domestic animals/pets at home? (a) Yes (b) No
16. What meat do you eat? Tick as appropriate a) pork meat b) bush meat c) chicken d) others-------
17. Have you ever used any drug by intravenous injection(non-medical use) (a) Yes (b) No
18. Is any member of the family/close family friend HIV positive? (a) Yes (b) No
19. Have you had sexual intercourse before? a) Yes b) No
20. If yes, are you homosexual? a) Yes b) No
21. Do you smoke tobacco? a) Yes b) No
22. Do you consume Alcoholic drinks? a) Yes b) No

**SECTION E: KNOWLEDGE OF HEPATITIS GENERALLY AND HEPATITIS E SPECIFICALLY**

1. Have you ever heard about hepatitis? a) Yes b) No
2. If yes, what is your source of information? a) TV/ radio/internet b) church c) school d) home e) others-----
3. What do you know about the modes of transmission of hepatitis? a) water-borne b) food c) blood transfusion c) direct contact d) others----- e) don’t know
4. What are the symptoms and signs? a) jaundice b) fever c) headache d)weakness e) others-
5. Have you ever heard about hepatitis E? a) Yes b) No
6. If yes, what is your source of information? a) TV/ radio/internet b) church c) school d) home e) others-----
7. What do you know about the modes of transmission of hepatitis E? a) water-borne b) food c) blood transfusion c) direct contact d) others----- e) don’t know
8. What are the symptoms and signs? a) jaundice b) fever c) headache d)weakness e) others-

**SECTION B: PROFORMA**

**PHYSICAL EXAMINATION FORM**

1. Code number-------------
2. Weight---------------
3. Height------------
4. Temperature-------------
5. General examination findings: a) pallor b) jaundice c) others-----
6. Digestive system a) abdominal tenderness b) hepatomegaly c) splenomegaly d) ascites e) others------

**LABORATORY TEST**

1. Anti-HEV IgG--------
